# Vegetable Nature of Diatomacæ

**Published:** 1869-12

**Authors:** 


VEGETABLE NATURE OF DIATOMACÆ.—Dr. H. L. Smith in the July number of the *American Journal of Science*, gives the results of his spectroscopic examination of the Diatomacæ, and comes to the conclusion that they are of vegetable nature. He has been able to prove the absolute identity of *chlorophyl* or the green endochrome of plants with *diatomin*

or the olive yellow endochrome of the Diatomacæ. By means of the spectrum-microscope, there is no difficulty in obtaining a characteristic spectrum from a living diatom, and to compare it directly with that of a desmid or other plant. From about fifty comparisons of spectra, Dr. Smith finds that the spectrum of chlorophyl is identical with that of diatomin. We refer to the original paper for a figure of the spectrum and for the precautions necessary to be observed in making the examination.—*Journal of Applied Chemistry.*

---

The following passages in the noble Inaugural Address of President Eliot at Harvard University attracts our special attention.

The statement is undoubtedly true which was uttered by Dr. Jacob Bigelow, that general scholarship, as the expression was understood and as that form of culture was attained fifty years ago, is now impossible; because the scope of each branch of knowledge has been so enlarged, and because so many new fields of research have been opened. Yet this statement is a good foundation stone for the additional block which President Eliot has hewn out for us to lay upon it the extract we now make.

"The actual problem to be solved is not what to teach, but how to teach. The revolutions accomplished in other fields of labor have a lesson for teachers. New England could not cut her hay with scythes, nor the West her wheat with sickles. When millions are to be fed where formerly there were but scores, the single fish-line must be replaced by seins and trawls, the human shoulders by steam elevators, and the wooden-axled ox-cart on a corduroy road by the smooth-running freight train. In education there is a great hungry multitude to be fed. The great well at Orvieto, up whose spiral paths files of donkeys painfully brought the sweet water in kegs, was an admirable construction in its day; but now we tap Fresh Pond in our chambers. The Orvieto might well remind some persons of educational methods not yet extinct. With good methods we may confidently hope to give young men of twenty or twenty-five an accurate general knowledge of all the main subjects of human interest, besides a minute and thorough knowledge of the one subject which each may select, as his principle occupation in life. To think this impossible is to despair of mankind; for unless a general acquaintance with many branches of knowledge, good as far as it goes, be attainable by great numbers of men, there can be no such thing as an intelligent public opinion; and in the modern world the intelligence of public opinion is the one condition of social progress."

"The examination [for admission to College] is conducted by college professors and tutors who have never had any relations whatever with those examined. It would be a great gain, if all subsequent college examinations could be as impartially conducted by competent examiners brought from without the college and paid for their services. When the teacher examines his class, there is no effective examination of the teacher. If the examinations for the scientific, theological, medical and dental degrees were conducted by independent boards of examiners, appointed by professional bodies of dignity and influence, the significance of these degrees would be greatly enhanced. The same might be said of the degree of Bachelor of Laws, were it not that this degree is, at present, earned by attendance alone, and not by attendance and examination."

We make one more brief extract:—

"The Medical Faculty affords another illustration of the same principle—that for real University progress we must look principally to the teaching bodies. The Medical School to-day is almost three times as strong as it was fifteen years ago. Its teaching power is greatly increased, and its methods have been much improved. The gain is the work of the Faculty of the School."—*Boston Med. and Surg. Journal.*

---

Dr. W. K. Bowling, of the *Nashville Journal of Medicine and Surgery*, is one of the few "jolly writers" on the solemn subjects of "physicians' fees, "ingratitude" of patients," "delinquent patrons, etc.," and draws "fun" from all such topics, as the bee sucks honey from the freshest clover blossoms. Read the following description of his method of "charging" his patients:

"'Do you charge anything for this?' said a substantial countryman, eyeing the hieroglyphics upon a prescription paper we had handed him, after we had thumped every rib in his body and every vertebra in his backbone, placed the bulb of a thermometer under his tongue and made a note of its revelation, and, with equal care and accuracy, secured the temperature of both axillæ; had placed a drop of his blood under a microscope of three hundred dollars' power, and written down what it said, besides, with gaslight, bull's-eye and laryngoscope illuminating his 'interior basin' down to his umbilicus; while all the time our steam atomizer was 'going it' at a white heat, its alcohol under a consuming fire, and the inevitable and everlasting carbolic acid going to waste, hissing, sighing and singing, and calling to mind the oft-repeated order of poor Barnaby Rudge's raven of 'Jenny put the kettle on, we'll all drink tea,' and all as if the unconscious machine was rejoicing in anticipation of a victim.

"'Charge?' we echoed, interogatively. 'No, sir, oh, no!' And we said it in a voice softened and modulated to the very sweetness of melody. (We had acquired the trick at an old-field la, me, fa, sol singing school when a boy.) 'Charge?' we repeated, with an intonation as dulcet as the musical fall of a dew drop from the willow into a silver basin, in the atmosphere of a Hannah Morean moon, whose light could be sliced by a golden knife. 'No, sir. We formerly did so; but the expense of book-keeping, and the added per cent. upon those who paid to secure us against losses from those who didn't, made the charging system (excuse us for a moment till we extinguish this lamp)—made the charging system a punishment to the honorable and a blessing to rogues, thus reversing a law of Heaven. No good man could be guilty of so revolting a sin after it was pointed out to him, and of course we quit charging. For our expenditure of skill upon your case this morning you only have to pay me ten dollars, which, under the abrogated and consequently obsolete rule of charging, would have been twenty. So you see, my dear sir, that you save ten dollars by this new system of charging nothing, and taken pay as we go. As he handed us the X, he said he had no doubt that the new rule was the best for all parties. 'Except the rogues,' said we. 'In course,' he assented, 'except the rogues.'"—*Jour. of Chemistry.*

